# Combination of fecal calprotectin and initial coronary dimensions to predict coronary artery lesions persistence in Kawasaki disease

**DOI:** 10.1038/s41598-022-12702-7

**Published:** 2022-05-23

**Authors:** Marianna Fabi, Emanuele Filice, Laura Andreozzi, Bianca Elisa Mattesini, Alessia Rizzello, Daniela Palleri, Elton Dajti, Rocco Maurizio Zagari, Marcello Lanari

**Affiliations:** 1 Pediatric Emergency Unit, Department of Medical and Surgical Sciences, IRCCS Azienda Ospedaliero-Universitaria, Polyclinic of St.Orsola, University of Bologna, 40138 Bologna, Italy; 2 Specialty School of Paediatrics, Alma Mater Studiorum, University of Bologna, 40138 Bologna, Italy; 3 Alma Mater Studiorum, University of Bologna, 40126 Bologna, Italy; 4 Pediatric Cardiology and Adult Congenital Heart Disease Program, Department of Cardio-Thoracic and Vascular Medicine, IRCCS Azienda Ospedaliero-Universitaria, Polyclinic of St.Orsola, University of Bologna, 40138 Bologna, Italy; 5 Gastroenterology Unit, IRCCS Azienda Ospedaliero-Universitaria, Polyclinic of St.Orsola, University of Bologna, 40138 Bologna, Italy

**Keywords:** Predictive markers, Paediatrics, Biomarkers, Cardiology, Risk factors, Cardiovascular diseases, Immunological disorders, Rheumatic diseases

## Abstract

Kawasaki Disease (KD) is systemic vasculitis involving medium-sized vessels in children. The aim of our study is to determine if fecal calprotectin (FC) could be useful in predicting the development or persistence of coronary artery lesions (CALs) in KD. We conducted a prospective monocentric study including all consecutive diagnoses of. Clinical, laboratory, echocardiographic data were recorded during the acute and subacute phase, including FC. Correlations among laboratory values, FC, clinical manifestations, IVIG-responsiveness and CALs development were investigated. We enrolled 26 children (76.9% boys; median age 34.5 months). The combination of FC > 250 microg/g and z-score > 2 during the acute phase was associated with the persistence of CALs (*p* = 0.022). A z-score > 2 alone during the acute phase was not related to CALs during the subacute stage (*p* > 0.05). A neutrophil percentage > 70% and WBC > 15,000/mmc during the acute phase significantly correlated with the presence of CALs during the subacute phase (*p* = 0.008). C-reactive protein (CRP) > 13 mg/dL at KD onset was significantly associated with the presence of CALs during the acute (*p* = 0.017) and subacute phase (*p* = 0.001). The combination of FC > 250 microg/g and a z-score > 2 during the acute phase of KD may be used as a predictor of CALs persistence. It can be useful especially in children with an initial CRP < 13 mg/dl.

## Introduction

Kawasaki disease (KD) is a necrotising arteritis involving medium-sized vessels mostly affecting children younger than 5 years of age. When coronary arteries are injured, severe cardiac complications, such as ischemic heart disease and sudden death^1^, can follow the disease, making KD the leading cause of pediatric acquired heart disease in high-income countries. The proper timing of the standard treatment with intravenous immunoglobulin (IVIG) is crucial to limit coronary lesions (CALs). An incomplete or atypical presentation of the disease can delay the diagnosis, thus increasing the risk of coronary involvement. Risk scoring systems to identify early those children at increased risk for severe forms have been validated in Asian children diagnosed with KD^2–5^, but they’re not predictive in non-Asian cohorts^6–8^.

Recently, Son et al. developed a risk score for CALs in a North American Cohort of children with KD, taking into account ethnicity, C-reactive protein (CRP), age and coronary involvement at the first ultrasound evaluation^9^.

Intestinal involvement in KD is frequent^10,11^ and, especially when prominent, it can be confusing, leading to a delay in the diagnosis potentially enhancing the risk of coronary involvement. Regardless of the severity of symptoms, abdominal manifestations have been shown to represent an independent risk factor for coronary aneurysms^11^. In KD mouse models, increased intestinal permeability and dysregulation of bowel immune system have been shown^12^.

Fecal Calprotectin (FC) is a marker of intestinal inflammation, widely used as an additional tool for diagnosis and management of Inflammatory Bowel Diseases (IBDs)^13–15^. FC is locally produced by activated granulocytes, mainly neutrophils^16^. Serum calprotectin (SC) has been found to be higher in KD patients during the acute phase compared to healthy subjects^17^. In addition, SC levels significantly decrease in IVIG-responders rather than in non-responder patients and persist in patients who developed giant aneurysms, or in adults who had previously suffered from myocardial infarction^17,18^.

To date, no data about fecal concentration of calprotectin in patients with KD are available.

The aim of our study was to evaluate whether FC during the acute phase of KD could help identifying more severe forms of disease.

## Results

A total of 30 patients were consecutively diagnosed with KD: 4 patients were excluded due to a late sample collection, and 26 patients (20 boys, 76.9%; median age 34.50 months, IQR 20.25–42.13 months) were enrolled. Of these, 18 (69.2%) were Caucasian, 7 (26.9%) were Asian, 1 (3.8%) was African.

According to the 2017 American Heart Association (AHA) definitions, 18/26 (69.2%) patients presented a complete form of KD, and 8/26 (30.8%) an incomplete form.

Twenty-two out of 26 (84.6%) patients were treated with IVIG at a median (IQR) time from onset of 8 (6–10) days: 18 (81.8%) were IVIG-responders, 4 (18.2%) were IVIG non-responders; 2/26 (7.7%) were late treated; 2/26 (7.7%) were not treated at all with IVIG. Laboratory tests of the acute stage of the disease are shown in Table 1.

**Table 1:** Laboratory values of patients diagnosed with KD enrolled in the study.

Parameter	Median (IQR)	Normal values
White Blood Cells (num × 10^9^/L)	15.0 (11.6–20.0)	4.8–12.0
Neutrophils (%)	73.7 (57.9–80.5)	33.0–74.0
Lymphocytes (%)	20.3 (13.0–30.0)	22.0–51.0
Red Blood Cells (num × 10^12^/L)	4.25 (4.02–4.56)	3.95–5.25
Platelets (num × 10^9^/L)	392.0 (320.0–480.8)	180.0–415.0
C-Reactive Protein (mg/dL)	8.99 (5.56–15.65)	< 0.5
Fecal Calprotectin (microg/g)	276 (105–898)	< 70
Interleukin 1beta (pg/mL)	1 (0–22.5)	< 5.9
Tumor Necrosis Factor alfa (pg/mL)	2 (0–11.5)	< 8.1 (reference value in adults)
Interleukin 6 (pg/mL)	71.4 (39.4–130.0)	< 4.7 (reference value in adults)
Interleukin 8 (pg/mL)	159.0 (27.0–13,844.0)	< 9.0
Total proteins (g/dL)	6.6 (6.4–7.0)	5.7–8.0
Albumin (g/L)	34.6 (32.5–38.8)	35.0–50.0
Sodium (mmol/L)	135.0 (133.3–137.0)	136.0–145.0
Aspartate aminotransferase (IU/L)	32.0 (29.0–47.8)	< 60.0
Alanine aminotransferase (IU/L)	22.0 (13.2–40.5)	< 45.0

Stool specimens for FC measurement were collected at a median (IQR) time from onset of fever of 6 (5–8) days. All patients had negative fecal occult blood test (FOBT), viral and bacterial fecal culture.

CALs developed in 11 (42.31%) patients during the acute phase (aneurysms: 8/11, 72.7%; dilations: 3/11, 27.3%), and persisted in 7 (26.9%) patients (aneurysms: 5/7, 71.4%, dilations: 2/7, 28.6%) during the subacute phase. A valvular regurgitation was documented in 9 (34.6%) patients and a pericardial effusion was present in 7 (26.9%) patients. Only 1 (3.8%) patient had a myocardial dysfunction.

White Blood Cells (WBC) > 15,000/mmc and neutrophils percentage > 70% correlated with CALs during the subacute (respectively *p* = 0.007 and *p* = 0.008), but not during the acute phase (*p* > 0.05).

CRP > 13 mg/dL significantly correlated with the presence of CALs during either the acute (*p* = 0.017) and subacute phase (*p* = 0.001). We did not find any significant correlation between the other laboratory values of the acute phase and CALs.

**Figure 1:**
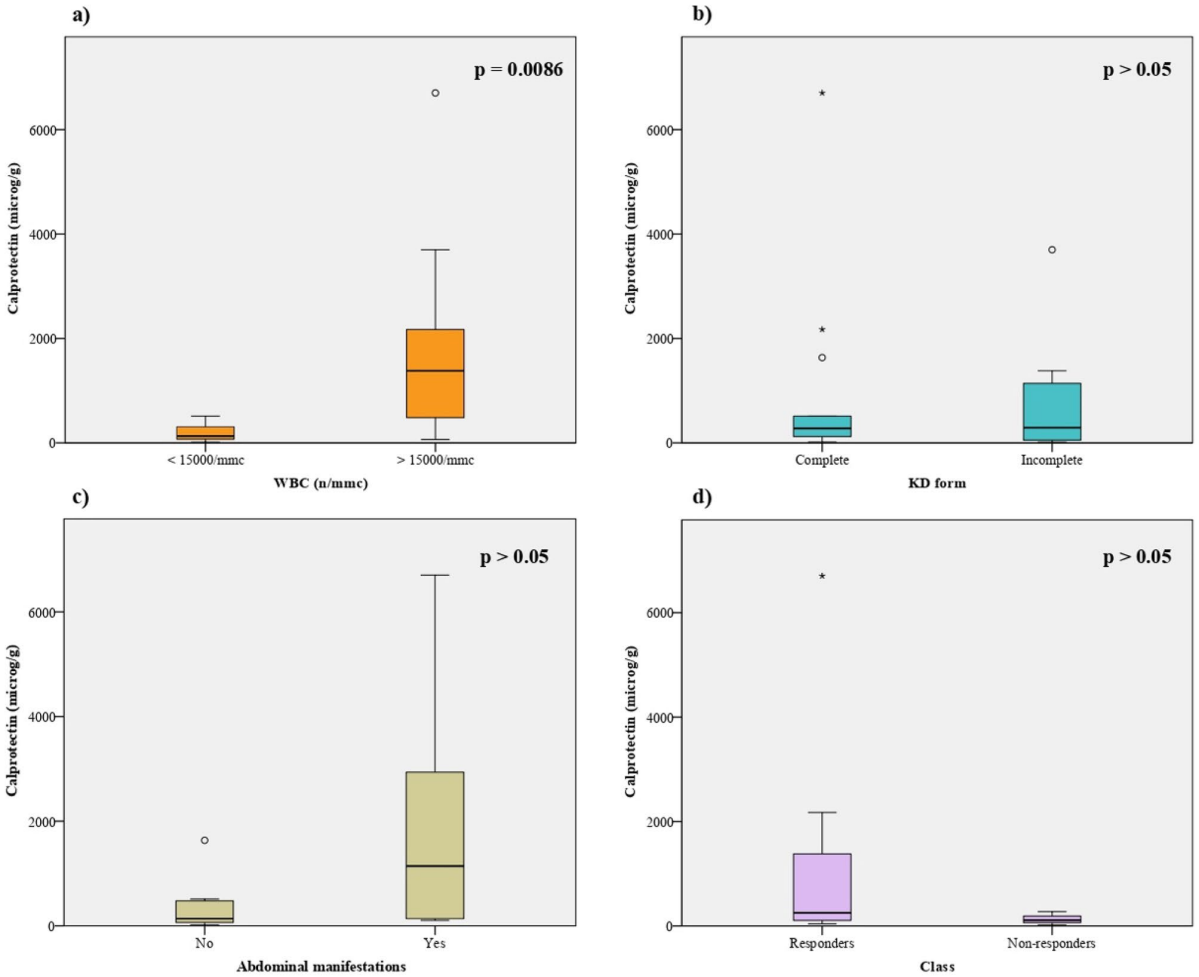
Comparison of FC concentration in patients with WBC > 15,000/mmc vs those with WBC < 15,000/mmc (**a**), in patients with abdominal manifestations vs those without (**b**), in IVIG-responders vs IVIG non-responders (**c**) and in patients with complete vs those with incomplete form (**d**). *Software: SPSS Statistics V25.*

FC was significantly higher in patients with WBC > 15,000/mmc (*p* = 0.0086), while it was higher but not significantly (*p* > 0.05) in patients with complete form compared to those with incomplete form, in IVIG-responders versus IVIG non-responders, and in patients presenting with abdominal manifestations versus those without (Fig. 1).

**Figure 2:**
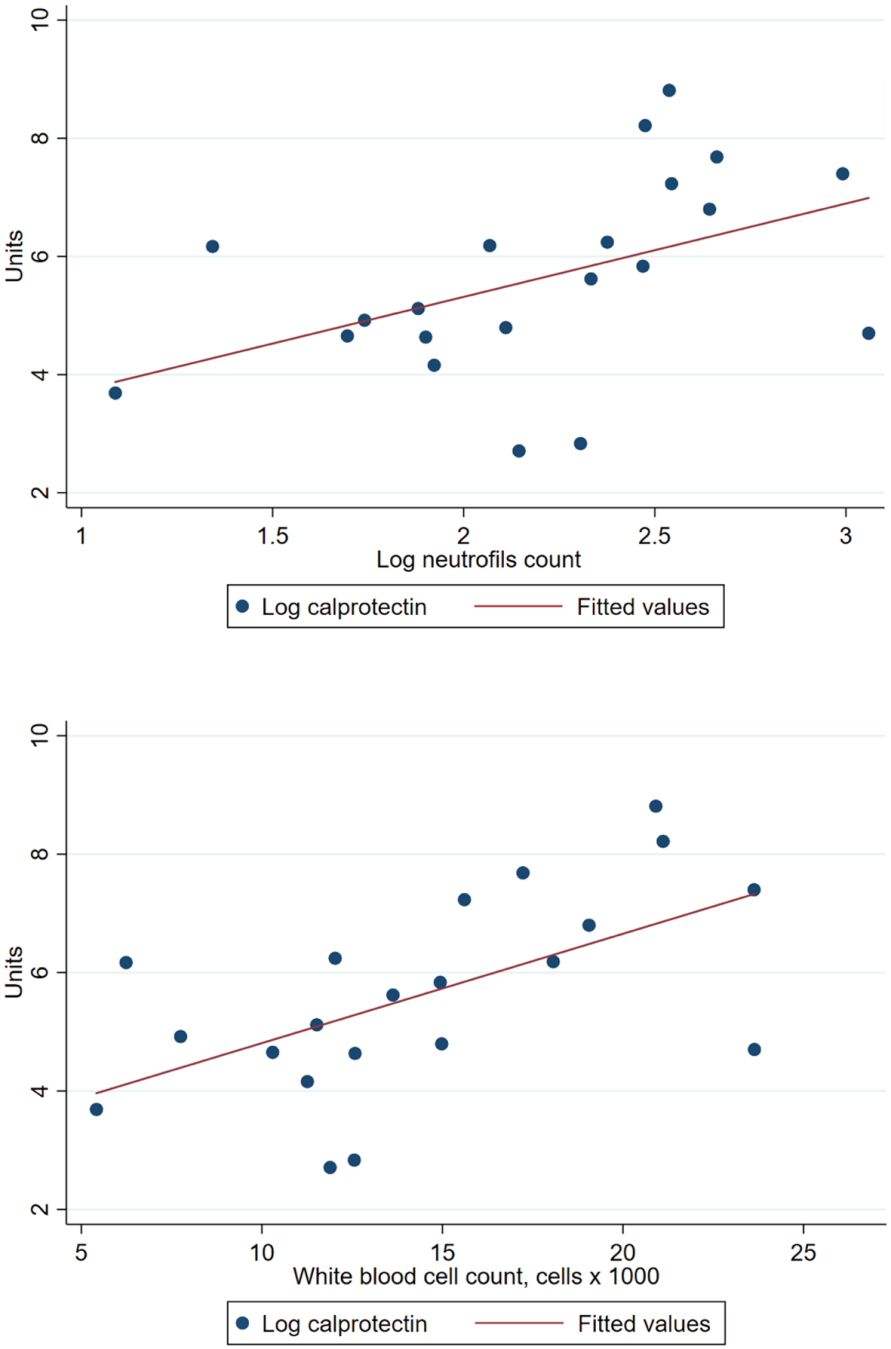
Correlation between fecal calprotectin concentrations and neutrophils count (slope = 1.57; intercept = 2.15) (above) and white blood cell count (slope = 0.18; intercept = 2.96) (below). A significant positive correlation was observed, respectively *p* = 0.03and *p* = 0.006. *Software: STATA 15*.

A significant positive correlation was shown between FC levels and WBC (*p* = 0.006) and between FC levels and absolute neutrophils count (*p* = 0.03) (Fig. 2).

We did not find any significant association between FC and a single clinical sign, IVIG-responsiveness, laboratory tests of the acute phase (neutrophils and lymphocytes percentage, hemoglobin, CRP, platelet count, aspartate aminotransferase, alanine aminotransferase, sodium, total proteins, albumin, cytokines), non-coronary cardiac lesions..

FC 70, FC 250, FC 500 alone were not associated with the presence of CALs neither in the acute phase nor in the subacute phase (*p* > 0.05). Similarly, a z-score > 2 during the acute phase had no correlation with the persistence of CALs later (*p* > 0.05).

Notably, the combination of FC 250 and z-score > 2 was predictive for persistence of CALs during the subacute phase (*p* = 0.022), while the combination FC 500 and z-score > 2 was not. Neutrophils percentage > 70% was not significantly correlated with CRP > 13 mg/dL, nor with FC.

## Discussion

Our findings show that FC values higher than 250 mcg/g in patients with a z-score > 2 during the acute phase of KD are associated with the persistence of coronary artery involvement during the subacute phase of the disease.

Four decades after the first description, KD is still an enigmatic disease diagnosed by the presence of clinical criteria without a pathognomonic marker. The most accurate way to assess this disease is the right interpretation of the combination of signs and symptoms suggestive for KD, supported by blood test results and echocardiographic abnormalities when present at diagnosis.

Despite their high frequency at onset, abdominal manifestations are nonspecific, and they are not included in the diagnostic criteria of the disease, but they have been linked to an increased risk for coronary artery aneurysms^11^. Gastrointestinal involvement could be due to bowel inflammation during the acute stage of KD: impairment of the intestinal barrier function and dysregulation of bowel immune system have been found in KD mouse models^19^.

FC is a well-known marker of intestinal inflammation, usually used in the diagnosis and monitoring of patients with IBDs, in association with other features^16,20^. It is a heterodimeric complex of S100A8 [calgranulin A or Myeloid Related Protein (MRP)-8] and S100A9 (calgranulin B or MRP-14) proteins^20^, released by activated neutrophils and monocytes at the sites of inflammation. Calprotectin is able to modulate cyclooxygenases activity^21^, to activate the innate immunity pathway and to favor leukocyte extravasation^22–24^. Paracrine and autocrine effects have been also described by inducing production of cytokines, matrix metalloproteinases, chemokines and reactive oxygen species. Thus, fecal levels depend on the migration of these cells, mainly neutrophils, to the inflamed bowel^25^.

SC is higher in patients with KD during the acute stage compared to healthy subjects, and rapidly decreases in responder patients after IVIG administration^17^. This finding could be related to the neutrophilic process affecting the coronary arteries during the acute phase of KD, that progressively destroys the vessel wall, resulting in CALs development^1^. In addition, SC levels persist high in patients, years after the diagnosis of KD, especially in those who developed giant aneurysms, as well as in those adults who had previously suffered from myocardial infarction^18^.

A more intense systemic inflammation is associated with a more severe disease and coronary injury in KD. Neutrophils and CRP values at the onset of the disease are associated with CALs and are included, indeed, in the risk score systems for either Asian and non-Asian children with KD^2–4,9,26^. In addition, neutrophils and monocytes/macrophages represent the main cells to be recruited in the coronary arterial wall during the acute stage of KD, playing a crucial role in the development of acute arteritis^27^. In our Cohort, a CRP higher than 13 mg/dL at onset was predictive for CALs during the acute and subacute phase. FC alone correlated with WBC and neutrophils, but not with CALs, and WBC and neutrophils in their turn correlated with the persistence of CALs. The lack of correlation between FC and CALs could be due to the small sample size.

On the opposite, the combination of FC > 250 mg/dL and a z-score > 2 at the initial echocardiogram, seems to identify children at higher risk for persistence of coronary involvement during the subacute stage who may benefit from an intensification treatment early in the course of the disease to limit coronary injury. This association could be particularly useful in patients with initial CRP levels lower than 13 mg/dl.

The main limitation of this study is the small sample size which is due to the low incidence of KD in our country and the monocentric design of the study.

In our geographic area, indeed, the incidence rate of KD is approximately 17.6 for 100,000 children under 5 years, much lower than the one reported in Asian countries^28^. Moreover, the sample size is partially due to the limited availability to measure fecal calprotectin in other hospital laboratories, contributing to the monocentric nature. On the other hand, our hospital protocol for KD could have minimized the possible variability on KD management.

An additional limitation could be the high variability of the FC cut-off according to age, diet and drugs^29^. To minimize the possible bias related to the variability of reference values, we decided to analyze 3 different published cut-off points for FC. Larger cohorts could help to define the more appropriate cut-off and to provide a new prognostic tool for coronary outcome, and, additionally, they could shed new light on the possible role of intestinal inflammation in the mechanism of KD. Our findings are preliminary, and a larger confirmatory study is needed. Future research should include the possible correlation between FC and SC in KD patients and the comparison of FC values among patients diagnosed with different childhood vasculitis.

In conclusion, this is the first study investigating a possible role of FC in the evaluation of patients diagnosed with KD. Our study shows that FC may be an additional tool to predict the risk of persistence of coronary lesions when combined with abnormal z-score at the initial evaluation. Since in our cohort an initial CRP level higher than 13 mg/dl was predictive for CALs during the acute and subacute phase, the relevance of our finding is emphasized in patients with initial CRP levels lower than 13 mg/dl. Further larger studies are needed to confirm our findings.

## Materials and methods

This was a monocentric prospective study including all children consecutively diagnosed with KD between January 2016 and May 2020 in the Pediatric Emergency Unit of Scientific Institute for Research and Healthcare (IRCCS) Policlinico di Sant'Orsola, Bologna, Italy.

All KD diagnoses were made in accordance with 2017 AHA Guidelines from expert pediatricians^1^.

Each patient underwent blood exams and echocardiography according to the methods described elsewhere^11^. Briefly, the acute stage was defined as the time from the onset of fever to the 10th day, and the subacute phase was from the 11th to the 20th day after onset. Inflammatory cytokines (interleukin 1beta, tumor necrosis factor alpha, interleukine 6, and interleukine 8) were also measured during the acute stage.

Coronary dimensions of the left anterior descending, circumflex and right coronary arteries were indexed by body mass index and expressed as z-scores: dilations and aneurysms were defined according to the AHA guidelines^1^. Non-coronary cardiac lesions were also considered, including mitral or aortic valvular regurgitation, ventricular dysfunction and pericardial effusion.

The main clinical features (fever, conjunctival hyperemia, lymphadenitis, mucosal abnormalities, skin rash and alterations of extremities), complete or incomplete clinical presentation, IVIG-responsiveness (i.e. the child was afebrile 36 h after the end of IVIG infusion) and late treatment (i.e. the child received the standard treatment after the 10th day from the fever onset), were documented and defined according to AHA^1^.

FC was measured in stool specimens collected within 10 days from disease onset and/or before IVIG infusion in case of late treatment. FOBT, bacterial stool cultures and viral antigen tests were collected at the same time in order to rule out other possible causes of increased FC.

FC was processed via Fluoro-Immuno-Enzymatic (FEIA) method; the cut off 70 mcg/g is the current normal reference level. FOBT was analyzed through the immunochemical method (iFOBT): a positive immunochemical test defined a positive test. Chemi-Luminescence Immuno Assays for detecting Rotavirus and Adenovirus antigen was performed in patient’s stool samples to exclude gastrointestinal viral infections. Stool cultures for Salmonella spp., Shigella spp. and Campylobacter spp. were done to rule out intestinal bacterial infections.

The study was approved by the local Ethics Committee (Comitato Etico Area Vasta Emilia Centro–AVEC, Bologna, Italy) (No. 98/2016/O/Sper). Written informed consent was obtained from the parents. The methodology in this study was in accordance with the relevant guidelines and regulations.

### Statistical analysis

Continuous variables were described with median and interquartile range (IQR), or mean and standard deviation (SD) if normally distributed, and categorical variables with percentages. Continuous data were compared using the Mann–Whitney test and categorical data using the Fisher exact test.

We analyzed 3 different cut-off levels for FC: 70 mcg/g (FC 70) as it is the current normal reference level; 250 mcg/g as FC levels of > 250 mcg/g (FC 250) have been suggested as cut-off values to predict mucosal inflammation in IBDs according to the European Society of Pediatric Gastroenterology, Hepatology and Nutrition (ESPGHAN)^29^, and 500 mcg/g (FC 500) to define very high values, often found in patients newly diagnosed with IBDs^30^. We analyzed one CRP cut-off level, that is > 13 mg/dl, as this was the cut-off found to predict risk for CALs development in a risk scoring system validated in a North American population with KD^9^.

The correlation between FC levels, using logarithm transformation, and continuous variables was assessed by linear regression analysis. A *p* < 0.05 was considered statistically significant. Statistical analyses were performed by STATA 15 statistical software (Stata Corp, College Station, Texas, USA).

### Ethics approval and consent

The study was approved by the local Ethics Committee (Comitato Etico Area Vasta Emilia Centro—AVEC, Bologna, Italy) (No. 98/2016/O/Sper).

### Consent for publication

Written informed consent for publication was collected from parents or legally authorized representative for each participant.

### Informed consent

Written informed consent was obtained from the parents.

## Data Availability

The datasets used and/or analysed during the current study are available from the corresponding author on reasonable request.
